# The Use of Agro-Food Chain By-Products and Foods of Plant Origin to Obtain High-Value-Added Foods

**DOI:** 10.3390/foods13193154

**Published:** 2024-10-03

**Authors:** Gianluca Nardone, Rosaria Viscecchia, Francesco Bimbo

**Affiliations:** Department of Agricultural Sciences, Food, Natural Resources and Engineering, University of Foggia, 71121 Foggia, Italyrosaria.viscecchia@unifg.it (R.V.)

The increased consumer demand for sustainable, health-promoting foods has propelled research into plant-based products and the valorization of food by-products. This Special Issue compiles pioneering research that focuses on the development of novel foods of plant origin and the incorporation of agro-food chain by-products into functional food products. These works not only address environmental concerns but also explore innovative ways to utilize underused resources [1,2].

Several articles in this Special Issue delve into the potential use of food by-products as valuable ingredients. Fredrick Nwude Eze et al. [Contribution 1] explore the upcycling of defatted sesame seed meal into protein amyloid-based nanostructures, showcasing its functional potential as a novel food ingredient. Similarly, Jordan Richards et al. [Contribution 2] investigate carrot pomace, a by-product of juice production, and the enhancement of its functional properties via physical treatments, thus contributing to waste reduction and an improvement in the sustainability of food. Victoria Baggi Mendonça Lauria and Luciano Paulino Silva [Contribution 3] further highlight how food residues can be transformed into natural colorants using green extraction methods, enhancing their stability through nanotechnology.

Research on plant-based products is also represented in this Special Issue. Spasoje D. Belošević et al. [Contribution 4] examine the phytochemical composition and antioxidant properties of cold-pressed microgreen juices, positioning them as rich sources of bioactive compounds for functional food markets. Nikoo Jabbari et al. [Contribution 5] optimize the extraction of bioactive compounds from saffron petals using ultrasound-assisted methods, demonstrating the value of saffron by-products in the production of natural antioxidants and pigments.

Contributing to the advancement of alternative protein sources, Kaitlyn Burghardt et al. [Contribution 6] investigate the thermal and rheological properties of spirulina, soy, pea, and brown rice proteins; they thus offer insights regarding the processing of plant-based meat substitutes, which is critical for reducing environmental harm. Sílvia Petronilho et al. [Contribution 7] analyze raspberry fruits discarded during dormancy, presenting strategies for their incorporation into food formulations while preserving their color and flavor. Papadopoulos et al. [Contribution 8] highlight the nutritional benefits of olive leaf extract in laying hens, demonstrating improvements in the quality of eggs and antioxidant parameters, which contribute to animal feed innovations that promote sustainability and enhance the quality of food. Similarly, Semeniuc et al. [Contribution 10] explore the presence of coenzyme Q10 in food by-products and waste, detecting it using high-performance liquid chromatography. This study underscores the potential of food by-products to be used as sources of valuable bioactive compounds. Additionally, Mariana-Atena Poiana et al. [Contribution 9] explore the integration of grape pomace into pastry formulations, revealing how the incorporation of by-products into functional food products can enhance functionality without compromising sensory appeal.

Sanad Alsbu et al. [Contribution 11] present an empirical model to predict the changes in the quality of fresh food during storage, providing a practical tool that can be employed by the food industry to reduce spoilage and food loss during storage. In a similar vein, Al-Naqeb et al. [Contribution 12] study the extraction of oils from cactus seeds by using supercritical fluid extraction, a technique that offers an efficient and sustainable method for extracting valuable components from agricultural by-products.

This Special Issue concludes with two reviews by Brennan et al. [Contribution 13] and Kong Yap and Al-Mutairi [Contribution 14]; these reviews explore regenerative food innovation and the role of agro-food chain by-products and foods of plant origin in obtaining high-value-added foods. They also discuss a conceptual model relationship between Industry 4.0, the food–agriculture nexus and the agroecosystem.

This collection of papers provides significant insights into how plant-based ingredients and by-products can contribute to sustainable food systems, highlighting the intersection of health, innovation, and environmental responsibility in the modern food industry [1,2].
